# Impact of Silver Nanoparticles on the Gut Microbiota of the Earthworm *Eisenia fetida*

**DOI:** 10.3390/ijms27020864

**Published:** 2026-01-15

**Authors:** Anita Zapałowska, Tadeusz Malewski, Andrzej Tomasz Skwiercz, Stanislaw Kaniszewski, Magdalena Muszyńska, Wojciech Hyk, Adam Masłoń

**Affiliations:** 1 Department of Agriculture and Waste Management, University of Rzeszów, St. Ćwiklinskiej 1a, 35-601 Rzeszów, Poland; 2 Department of Molecular and Biometric Techniques, Museum and Institute of Zoology, Polish Academy of Sciences, 00-818 Warsaw, Poland; tmalewski@miiz.waw.pl; 3 The National Institute of Horticultural Research, 96-100 Skierniewice, Poland; andrzej.skwiercz@inhort.pl (A.T.S.); stanislaw.kaniszewski@inhort.pl (S.K.); 4 Faculty of Chemistry, University of Warsaw, 02-093 Warsaw, Poland; magdalena.muszynska@pepolska.pl (M.M.); wojhyk@chem.uw.edu.pl (W.H.); 5 Department of Environmental Engineering and Chemistry, Rzeszow University of Technology, 35-959 Rzeszów, Poland; amaslon@prz.edu.pl

**Keywords:** bacterial diversity, 16S rRNA gene, AgNP exposure

## Abstract

Silver nanoparticles (AgNPs) are increasingly applied in agriculture and related technologies due to their antimicrobial properties, yet their interactions with soil-associated organisms and microbial communities remain insufficiently characterized. This study examined the effects of AgNP exposure (10.85 mg/L) on trace element accumulation and gut bacterial communities of the earthworm *Eisenia fetida* under two substrate conditions (horticultural substrate and compost). High-throughput 16S rRNA gene sequencing revealed substrate-dependent shifts in microbial community structure following AgNP exposure. Several bacterial taxa, including *Proteobacteria*, *Gammaproteobacteria*, *Bacilli*, *Streptococcus* sp., and *Staphylococcus* sp., exhibited pronounced numerical declines, indicating sensitivity to AgNPs, whereas *Actinobacteria* and *Bacteroidetes* showed comparatively higher relative abundances, suggesting greater tolerance. Compost partially mitigated the inhibitory effects of AgNPs on gut microbiota. Concurrently, AgNP exposure altered trace element accumulation patterns in earthworm tissues, highlighting interactions between silver uptake and elemental homeostasis. Collectively, these findings demonstrate that AgNPs can induce taxon- and substrate-specific responses in earthworm-associated microbial communities and metal accumulation, providing insight into potential ecological consequences of nanoparticle use in agricultural systems.

## 1. Introduction

Modern agriculture is increasingly turning to nanotechnology, including silver na-noparticles (AgNPs), due to their potent antimicrobial properties. AgNPs are utilized as plant protection agents against fungal and bacterial diseases [1,2,3], components of nano-fertilizers [4], and coatings to extend the shelf life of agricultural products [5,6].

Owing to their multifunctionality, AgNPs hold significant potential as plant pro-tectants, growth stimulators, nano-fertilizer additives, and agents for improving crop preservation and quality. However, due to potential adverse effects on the environment and human health, thorough research on the safety of AgNP application is essential. In particular, understanding the environmental fate of metallic AgNPs is critical, as they are largely transformed into silver sulfides (Ag_2_S) during wastewater transport and treatment processes [7,8,9,10,11,12,13,14].

Additionally, sewage sludge, which may contain these transformed nanoparticles, is commonly applied as fertilizer in agriculture [15,16,17].

Recent studies have shown that nanoparticles are to some extent bioavailable and can cause toxic effects in various organisms [18,19,20,21]. Once released into the environment, they can come into contact with organisms and accumulate in their bodies [22,23,24,25,26,27,28]. Studies have shown that silver ions are more toxic than silver nanoparticles. In both cases, toxicity increases over time, leading to reduced reproduction rates in juvenile organisms and in-creased mortality [29].

Earthworms are widely recognized as effective bioindicators of metal contamination in soils due to their capacity to absorb and accumulate various metals from their environment. Their burrowing and feeding activities result in the continuous ingestion and absorption of soil particles and associated contaminants, including heavy metals such as cadmium (Cd), lead (Pb), and zinc (Zn). The bioaccumulation of metals in earthworm tis-sues reflects the bioavailable fraction of metals in the soil environment rather than total metal concentrations, providing a more ecologically relevant measure of contamination. Additionally, earthworms possess physiological mechanisms that regulate metal uptake, storage, and detoxification, such as metallothionein proteins, which facilitate the sequestration of metals in their tissues. Consequently, earthworm metal concentrations serve as reliable biomarkers for monitoring spatial and temporal variations in soil metal pollution, contributing to environmental risk assessment and soil health evaluation. Species such as *Eisenia fetida* are widely used as model organisms in toxicological risk assessments [30,31]. As bioindicators of soil health, *E. fetida* plays crucial role in agroecosystems, including the decomposition of organic matter, enhancement of soil structure, stimulation of beneficial microbial activity, and support of nutrient cycling [32] and bioremediation [33,34].

The aim of this study was to expand current knowledge on the toxicity of silver nanoparticles (AgNPs) to the soil model organism *E. fetida*, with particular emphasis on their bioavailability, bioaccumulation, and potential sublethal effects under conditions approximating an agricultural environment. The results obtained provide a deeper understanding of the interactions between transformed forms of silver, such as those found in sewage sludge, and soil organisms, which may contribute to more accurate assessments of the environmental risks associated with the use of nanomaterials in agriculture.

## 2. Results

### 2.1. Trace Element’s Concentration in E. fetida Tissues

In our experiment, Tukey’s HSD test showed statistically significant differences in silver concentrations in the tissues of *Eisenia fetida* between groups and across variants.

**Figure 1:**
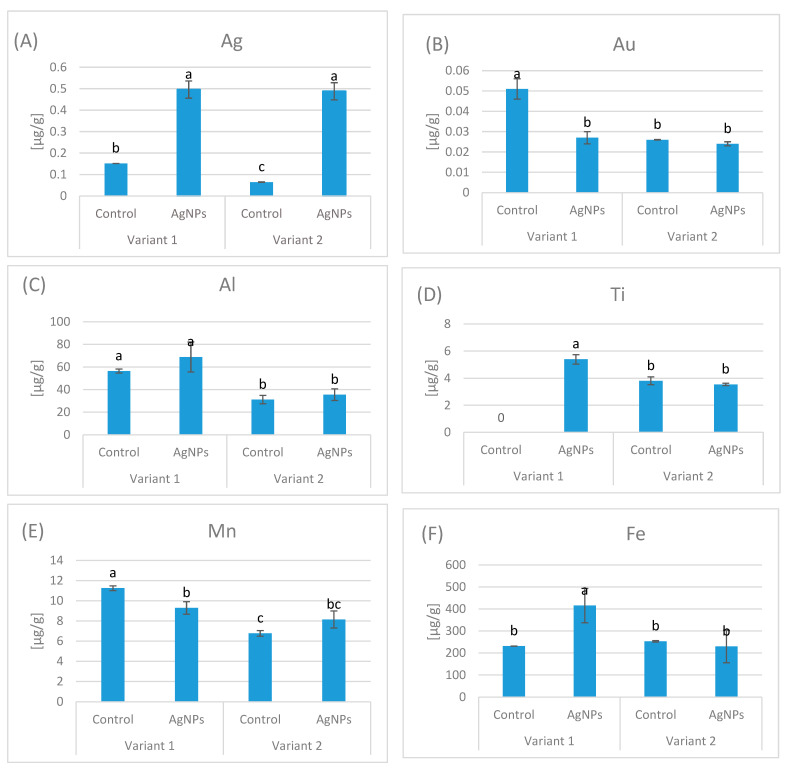

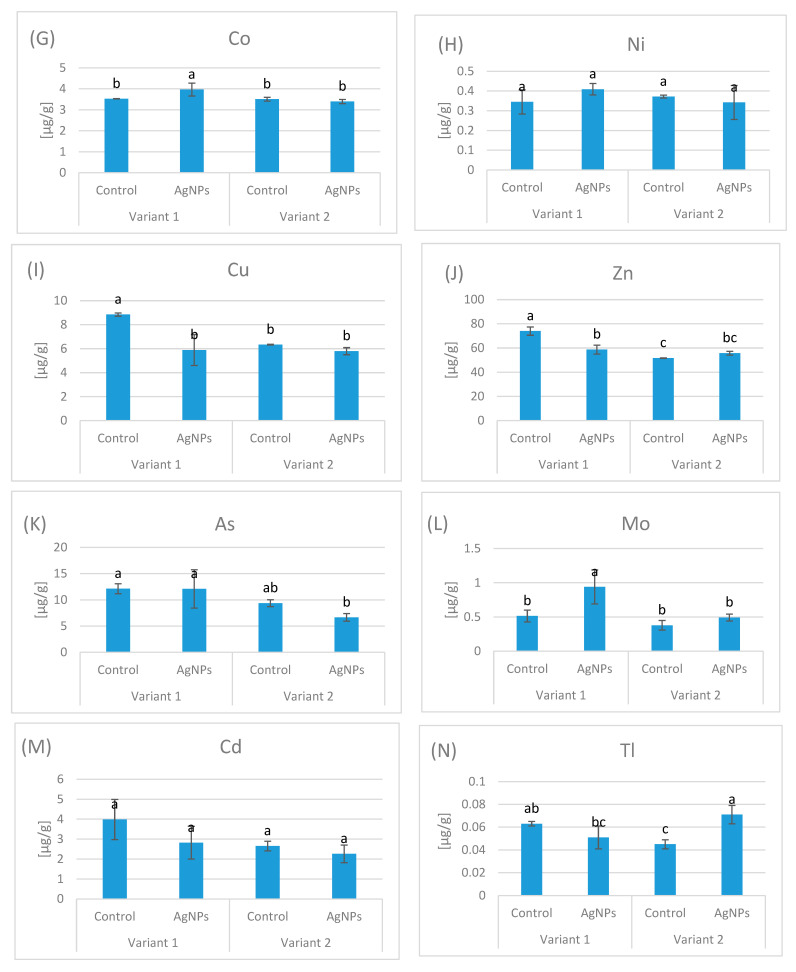

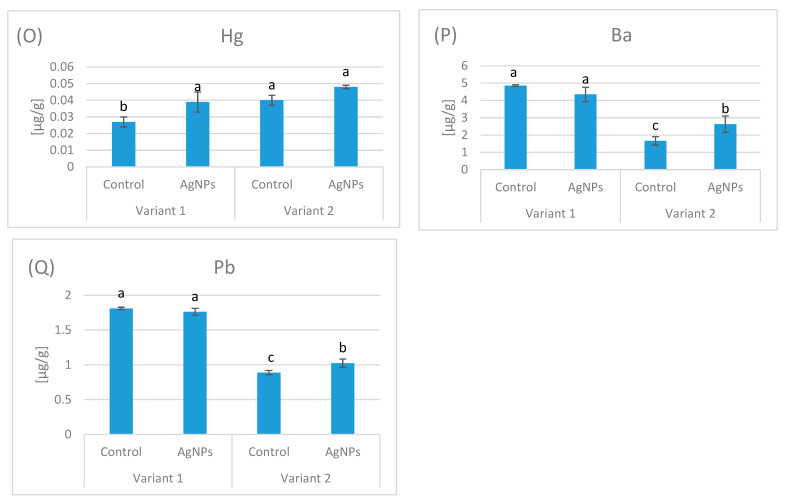
Comparison of metal concentrations Ag (**A**), Au (**B**), Al (**C**), Ti (**D**), Mn (**E**), Fe (**F**), Co (**G**), Ni (**H**), Cu (**I**), Zn (**J**), As (**K**), Mo (**L**), Cd (**M**), Tl (**N**), Hg (**O**), Ba (**P**), Pb (**Q**) in control and AgNPs-treated samples for Variant 1 and Variant 2. Different letters indicate significant differences (*p* < 0.05), (*n* = 3). Bars represent standard deviation (SD).

In Variant 1, the control group, despite not being exposed to silver, showed a background tissue Ag level of 0.152 µg/g, whereas the AgNPs-treated group exhibited a markedly higher concentration of 0.496 µg/g. Similarly, in Variant 2, the control group displayed only a trace background tissue concentration of 0.065 µg/g, while the AgNP-exposed group reached 0.488 µg/g (Figure 1A).

In Variant 1, several metals exhibited notable increases following AgNP exposure, including Fe (+80%), Mo (+83%), and Hg (+44%). In addition, Ti, which was below the detection limit in the control, became detectable after AgNPs treatment. In contrast, the concentrations of Au (−47%), Mn (−17%), Cu (−33%), Zn (−21%), Cd (−29%), and Tl (−19%) decreased, indicating an inhibitory effect of AgNPs on the accumulation of certain elements in this substrate.

In Variant 2, responses were generally less pronounced or opposite in direction compared to Variant 1. Metals such as Al (+14%), Mn (+20%), Ba (+58%), Hg (+20%), Tl (+58%), Mo (+30%), and Pb (+15%) increased under AgNPs treatment, whereas Fe (−9%), Co (−15%), Cu (−8%), As (−29%), and Ti (−7%) showed decreases. Notably, Variant 2 showed a reduced inhibitory effect overall, suggesting that the substrate type modulates the impact of AgNPs on metal concentrations (Figure 1B–Q)

**Figure 2:**
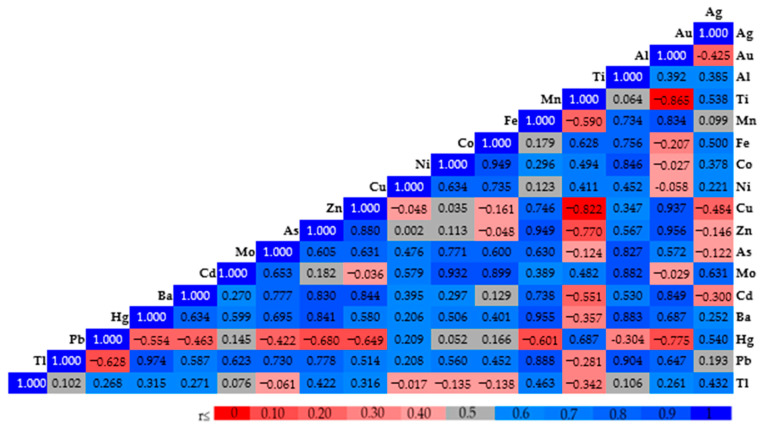
Pearson correlation coefficients between the selected parameters of the analyzed traits. Correlation coefficient (r) scale: −1.0: perfect negative correlation; −0.80 to −0.60: strong negative correlation; −0.40 to −0.20: moderate negative correlation; 0: no correlation; 0.20 to 0.40: moderate positive correlation; 0.60 to 0.80: strong positive correlation; 1.0: perfect positive correlation.

As shown in Figure 2, selected correlations highlight the relationships between silver nanoparticle (AgNPs) exposure and specific heavy metals. Ag were negatively correlated with Au (r = −0.425), Cu (r = −0.484), and Cd (r = −0.300). Conversely, Ag showed positive correlations with Mo (r = 0.631) and Hg (r = 0.540).

### 2.2. Bacterial Community Abundance

**Figure 3:**
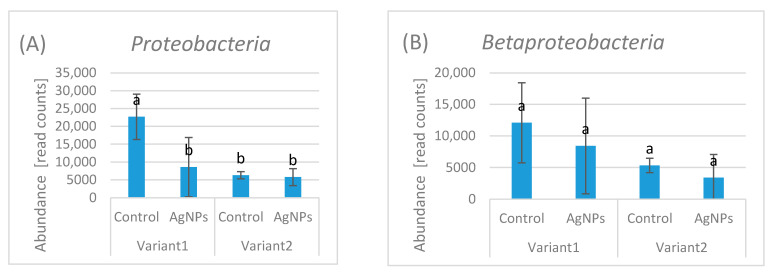

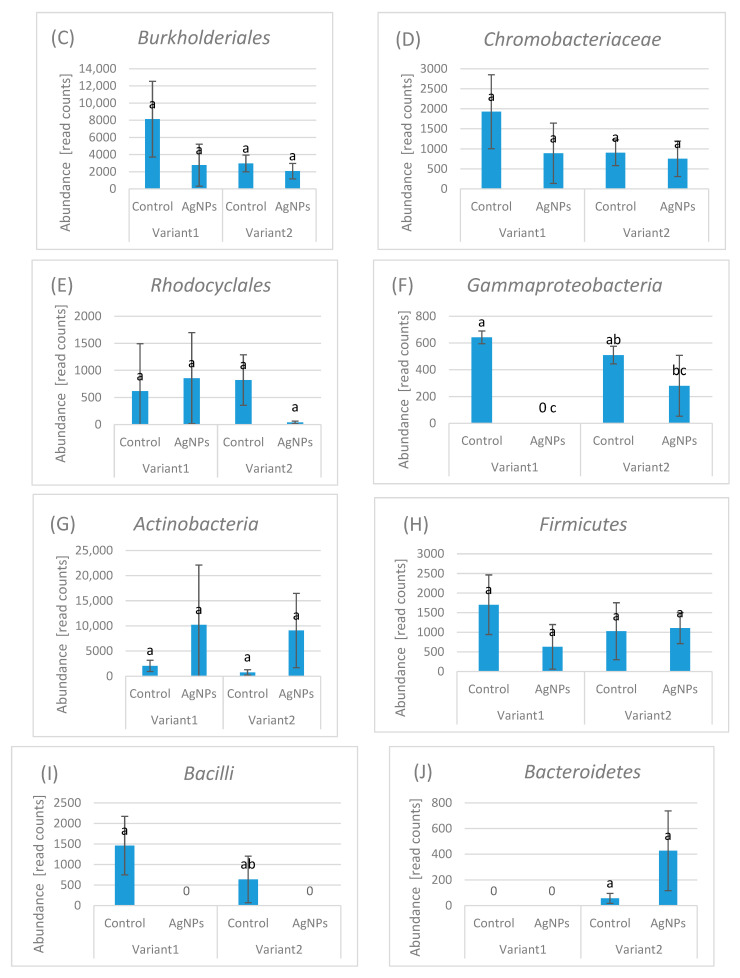

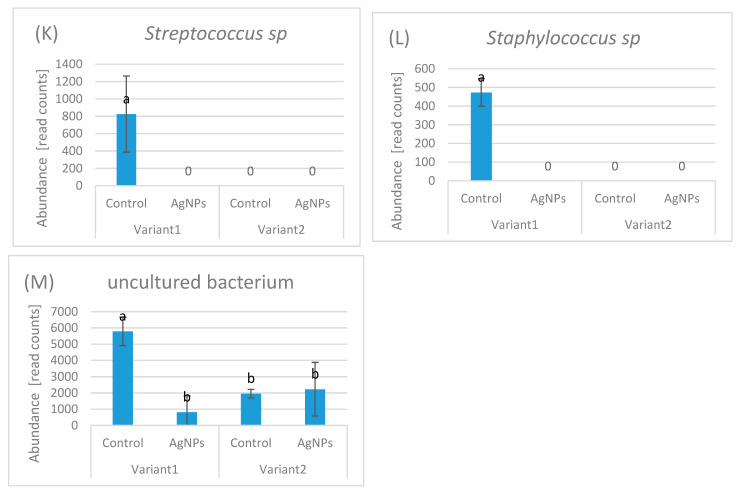
(**A**–**M**) Abundance of selected bacterial taxa under control and AgNPs treatments in Variant 1 (substrate) and Variant 2 (compost). Data are shown as mean read counts. Different letters indicate significant differences (*p* < 0.05), (*n* = 3). Bars represent standard deviation (SD).

*Proteobacteria* abundance varied between variants and treatments. In Variant 1 (substrate), the control showed 22,684 read counts, whereas AgNP exposure reduced abundance to 8578 read counts, representing a 62% decrease relative to the control. In Variant 2 (compost), control abundance was 6304 read counts and AgNPs treatment slightly reduced it to 5779 read counts (8% decrease), demonstrating minimal impact in this substrate (Figure 3A).

*Betaproteobacteria* abundance showed a decreasing trend across treatments, with read counts of 12,095.5, 8424, 5330, and 3389.3, respectively. The lowest value represents a 72% decrease relative to the highest, but statistical analysis indicated no significant differences among treatments (Figure 3B).

Similarly, *Burkholderiales* abundance ranged from 8118.5 in Variant 1 Control to 2080.3 in Variant 2 AgNPs, a 74% decrease, yet no significant differences were detected between groups (Figure 3C).

*Chromobacteriaceae* abundance varied across the experimental conditions, with read counts of 1927, 889, 905, and 752. The lowest value represents a 61% decrease relative to the highest. Despite this apparent decline, statistical analysis indicated no significant differences between the groups (Figure 3D).

*Rhodocyclales* abundance varied among experimental conditions, with read counts of 619, 858, 823, and 42, respectively. Although individual values varied considerably, the treatments did not have a statistically detectable effect on *Rhodocyclales* abundance (Figure 3E).

Notably, after exposure to AgNPs *Gammaproteobacteria* were not detected in AgNPs-treated samples of Variant 1, suggesting a strong negative response; however, absence of reads does not necessarily indicate complete biological elimination (Figure 3F). Although rarefaction curves indicated adequate sequencing depth, zero read counts should not be interpreted as definitive biological absence, as they may reflect detection limits or compositional effects. Large numerical differences that did not reach statistical significance should be interpreted cautiously, as high inter-replicate variability and limited sample size (n = 3) reduce statistical power.

*Actinobacteria* abundance fluctuated across treatments, including AgNP exposure, these differences were not statistically detectable (Figure 3G).

In *Firmicutes*, AgNP exposure resulted in a 63% reduction in Variant 1, whereas Variant 2 showed a 7.7% increase relative to the control (Figure 3H).

Similarly to *Gammaproteobacteria Bacilli* were not detected in AgNPs-treated samples under the applied sequencing depth in both variants, also suggesting a strong negative response (Figure 3I).

*Bacteroidetes* abundance was very low across treatments. Read counts ranged from 0 to 427, with the highest value observed in one AgNPs-treated sample (427 read counts), suggesting minimal numerical variation. Statistical analysis indicated no significant differences among group (Figure 3J).

Both *Streptococcus* sp. and *Staphylococcus* sp. showed extreme sensitivity to AgNP exposure. In *Streptococcus* sp., read counts were 825 in the control and completely reduced to 0 in Variant 1. Similarly, *Staphylococcus* sp. decreased from 473 read counts in the control to 0 under Variant 1 conditions, demonstrating complete suppression. Both *Streptococcus* sp. and *Staphylococcus* sp. were completely absent in Variant 2 (compost) (Figure 3K,L).

In Variant 1, uncultured bacteria abundance ranged from 815 to 5785 read counts, corresponding to an 86% decrease relative to the maximum observed value (Figure 3M).

**Figure 4:**
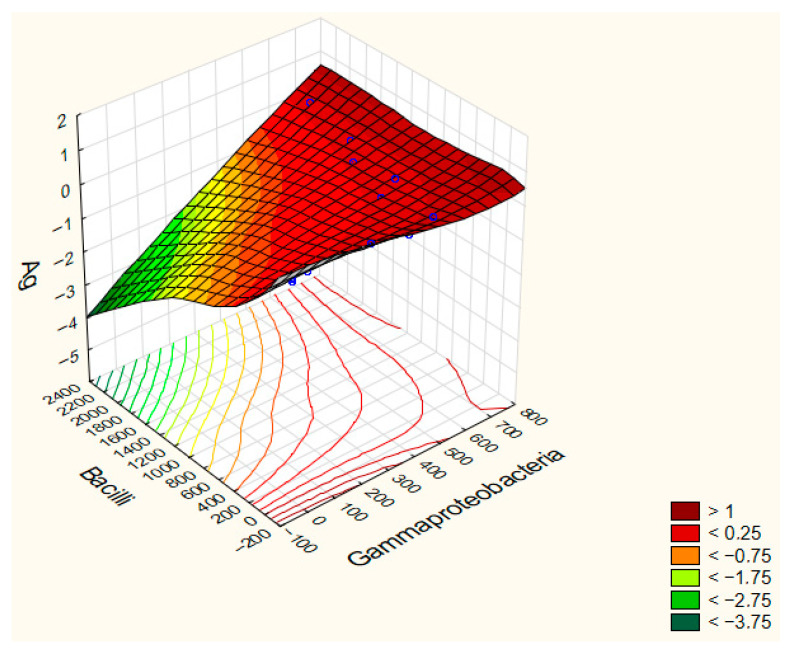
Surface regression model plots for Ag, *Bacilli*, and *Gammaproteobacteria*. Blue dots in the graph represent the experimental measurements.

A strong negative correlation was observed between *Gammaproteobacteria* abundance and AgNP exposure (r = −0.822), indicating that concentrations of AgNPs were associated with lower *Gammaproteobacteria* counts. Similarly, *Bacilli* abundance showed a moderate negative correlation with AgNPs (r = −0.663), demonstrating a comparable, though slightly weaker, inhibitory effect of silver nanoparticles on this bacterial group (Figure 4).

Correlation analysis revealed variable relationships between AgNP exposure and bacterial abundance across different taxa. Negative correlations were observed for most groups, indicating that AgNPs concentrations were associated with lower abundance: *Proteobacteria* (r = −0.315), *Betaproteobacteria* (r = −0.247), *Burkholderiales* (r = −0.420), *Chromobacteriaceae* (r = −0.389), *Rhodocyclales* (r = −0.374), *Gammaproteobacteria* (r = −0.822), *Firmicutes* (r = −0.380), *Streptococcus* sp. (r = −0.390), *Staphylococcus* sp. (r = −0.431), *Bacilli* (r = −0.664), and uncultured bacteria (r = −0.494).

In contrast, positive correlations were observed for *Actinobacteria* (r = 0.558) and *Bacteroidetes* (r = 0.333), demonstrating that AgNP exposure was associated with increased abundance in these groups. These selected correlations indicate that AgNP exert differential effects on bacterial communities, with most groups being inhibited while a few taxa, such as *Actinobacteria* and *Bacteroidetes*, potentially benefit or tolerate the presence of nanoparticles.

The variability observed among biological replicates likely reflects the inherent heterogeneity of earthworm gut microbiota and individual-level differences in feeding activity, gut transit time, and microhabitat interactions within the substrate. Additionally, microbial community data based on relative abundance are sensitive to compositional effects, particularly in small sample sizes, where changes in dominant taxa can amplify apparent variability among replicates. Such variability is commonly reported in gut microbiome studies of soil invertebrates and does not necessarily indicate experimental inconsistency but rather biological realism under environmentally relevant exposure conditions.

## 3. Discussion

Exposure to silver nanoparticles (AgNPs) significantly influenced the concentrations of trace elements in *Eisenia fetida* tissues across both experimental variants.

Substrate type played a central role in modulating the interactions between AgNP exposure, gut microbiota composition, and trace element balance in *E. fetida.* The compost substrate, characterized by higher organic matter content and nutrient availability, likely reduced AgNPs bioavailability through adsorption, complexation with humic substances, and enhanced microbial immobilization. These processes may limit nanoparticle–microbe contact and mitigate ionic silver release, resulting in attenuated microbial disruption and more stable elemental profiles compared to the standard substrate. This substrate-mediated buffering effect represents an understudied pathway through which soil properties influence nanoparticle toxicity in agroecosystems.

Statistically significant differences were observed for elements such as Au, Fe, Cu, Zn, Mo, Cd, Ba, and Pb, which are either essential micronutrients or known environmental contaminants involved in biological or toxicological processes.

The accumulation of silver (Ag) was accompanied by a decrease in copper (Cu) accumulation. Copper is an essential element that is specifically stored by metallothioneins- stress proteins that bind both essential and toxic metals through thiol bonds. Metallothioneins play a crucial role in maintaining the homeostasis of essential metals such as zinc (Zn) and copper (Cu), as well as toxic metals like cadmium (Cd) and mercury (Hg) [35,36,37,38,39,40,41,42,43,44,45,46,47].

Iron (Fe) and copper (Cu) are key players in cellular respiration and the regulation of oxidative stress. A study by Zhang et al. [38] demonstrated that in soils with elevated Cu concentrations, antioxidant enzymes in *Eisenia fetida* were inhibited, indicating an intense oxidative-stress response that simultaneously reduced cellular energy efficiency.

Zinc (Zn) is vital for enzyme activity and protein function [39], while molybdenum (Mo) serves as a cofactor in redox reactions [40]. In contrast, cadmium (Cd), barium (Ba), and lead (Pb) are non-essential toxic metals known to interfere with metabolic pathways [41].

The observed changes demonstrate that AgNP exposure may disrupt metal accumulation mechanisms and compromise elemental homeostasis in *Eisenia fetida* tissues. In contrast, elements such as Mn, Ni, and Hg showed no significant changes, indicating selective or limited interactions under the tested conditions (Figure 1).

Numerous studies have documented metal accumulation in earthworms, forming a substantial body of research. The majority of these investigations have concentrated on specific metals such as cadmium (Cd) [42,43,44], copper (Cu), zinc (Zn), lead (Pb) [45,46,47], nickel (Ni) [48] mercury (Hg) [49] and arsenic (As) [46,50]. Fewer studies have examined the accumulation of manganese (Mn) [45] and chromium (Cr) [51]. Additionally, researchers have explored the relationship between metal accumulation in earthworm tissues and exposure dose [49,51,52].

The concentration levels of the nanoparticle formulations used in this study were deliberately selected based on prior experimental findings. In our earlier research involving the same nanoparticle formulations, these concentrations proved to be optimal for eliciting a measurable biostimulatory effect in plants, while avoiding adverse outcomes associated with elemental accumulation. Consequently, a supplementary objective of the present study was to assess whether the application of these formulations might exert negative effects on non-target soil organisms.

In Kim et al.’s study [53], 12 bacterial groups were isolated from the gut of *Eisenia fetida* inhabiting contaminated soil, with *Firmicutes*—particularly *Bacillus* spp.—being the most dominant (31%), followed by *Rhodococcus* (19%) and *Pseudomonas* (10%), which are common soil- and gut-associated bacteria.

In our experiment, Variant 1 control samples revealed a dominance of *Proteobacteria* (100%), with *Betaproteobacteria* and *Burkholderiales* prevailing, and only a minor representation of *Firmicutes* (4.28%), including genera such as *Streptococcus* and *Staphylococcus*. Compared to Valle-Molinares et al. [54], who identified multiple *Bacillus* species from *Onychochaeta boricana*, our findings reveal a marked suppression of *Bacilli* in AgNPs-treated samples, where they were entirely absent. Bacteria like *Aeromonas*, *Pseudomonas*, and *Acinetobacter*, which were reported by Brito et al. [55] as common in *Pontoscolex corethrurus*, were not prominent in our dataset.

Soil microorganisms play a critical role in organic matter decomposition and nutrient cycling [56,57]. Bacteria metabolize the resulting simpler compounds. They also form a natural component of the earthworm diet, and their diversity within the gut is closely associated with the efficiency of decomposition processes and the availability of nutrients for plants. The introduction of silver nanoparticles (AgNPs) into soil ecosystems has been shown to profoundly affect microbial communities due to their potent antimicrobial properties. AgNPs toxicity operates primarily through two mechanisms: (1) the generation of reactive oxygen species (ROS), such as superoxide anions (O_2_^−^), on the nanoparticle surface, and (2) the release of silver ions (Ag^+^), which bind to thiol groups in essential enzymes and proteins, disrupting cellular respiration and mem-brane integrity [18,58,59,60]. These effects are modulated by nanoparticle characteristics (size, shape, surface coating) and soil properties. In our study, among all taxa, *Gammaproteobacteria* and *Bacilli* showed the strongest negative correlations, highlighting their particular sensitivity to AgNP exposure.

Although microbial communities are ecologically significant and highly responsive to environmental disturbances, detailed insights into the impact of silver nanoparticles (AgNPs) on soil microbiota remain limited [61]. Numerous studies documented the dif-fering physico- chemical and concomitant toxicological behavior of AgNPs in dependence of the soil type [62,63].

The study by Grün et al. [63] demonstrated that the impact of silver nanoparticles (AgNPs) on soil microbial communities depends on their functionalization, concentration, exposure time, and soil texture. *E. fetida* earthworms were exposed for 10 days to soils amended with 2, 10, and 50 mg/kg of silver, administered as either nanoparticles (AgNPs) or ionic silver. Exposure to AgNPs induced oxidative stress, as indicated by elevated glutathione S-transferase activity, reduced catalase activity, and increased malondialdehyde levels. Notably, protein carbonylation served to distinguish nanoparticulate from ionic silver, with significant increases observed only at the highest nanoparticle concentrations. DNA damage was evident even at the lowest AgNPs concentration, and at higher levels, both nanoparticulate and ionic silver forms downregulated the expression of genes associated with general defense and stress responses. Despite their lower bioavailability relative to ionic silver, AgNPs demonstrated comparable or even greater toxicity when accumulated in earthworm tissues at similar concentrations.

Earthworm gut microbiota contribute to key agroecological functions, including organic matter decomposition, nutrient mineralization, and microbial inoculation of soils through cast production. Consequently, AgNPs induced shifts in gut bacterial communities- particularly reductions in taxa associated with decomposition and nutrient cycling- may indirectly influence soil fertility, microbial resilience, and ecosystem functioning. Although microbial functional activity was not directly assessed in this study, the observed taxonomic responses suggest that nanoparticle exposure may affect agroecosystem processes mediated by earthworms, extending the relevance of this work beyond basic toxicity assessment.

This study focused on taxonomic composition of gut bacterial communities based on 16S rRNA gene sequencing. No functional prediction or metagenomic profiling was conducted; therefore, observed taxonomic shifts cannot be directly translated into changes in microbial metabolic or ecological functions. Future studies integrating functional prediction tools or shotgun metagenomics would be necessary to establish mechanistic links between AgNP exposure, microbial function, and host ecological performance.

## 4. Materials and Methods

### 4.1. Experiment Design

The experiment was conducted in the laboratory of the Institute of Horticulture in Skierniewice (51°96′15″ N, 20°13′69″ E) between 20 March and 14 April 2025. The aim of the study was to compare the effects of silver nanoparticles (AgNPs) on selected biological parameters of the earthworm *Eisenia fetida*. Two substrate variants were used in the experiment:

1. Variant 1: Standard horticultural substrate by Klasmann (Geeste, Germany), ProLine Potgrond line, commonly used in organic cultivation. This substrate was composed of high-moor peat (H5–H8) with the following chemical characteristics: nitrogen content 350–450 mg·kg^−1^, phosphorus pentoxide 250–350 mg·kg^−1^, potassium oxide 350–500 mg·kg^−1^, magnesium oxide 100–200 mg·kg^−1^, salt content 1.0–1.8 mg·kg^−1^, and pH (H_2_O) in the range of 5.5–6.5.
2. Variant 2: Compost obtained from horticultural and household organic waste. The final chemical composition of the compost was as follows: neutral pH of 6.8, electrical conductivity of 2.9 mS·cm^−1^. The macro- and micronutrient profile included: nitrates (215 mg·kg^−1^), ammonium (47 mg·kg^−1^), phosphorus (308 mg·kg^−1^), potassium (746 mg·kg^−1^), calcium (1763 mg·kg^−1^), magnesium (234 mg·kg^−1^), sodium (259 mg·kg^−1^), sulfates (156 mg·kg^−1^), chloride (282 mg·kg^−1^), iron (50.8 mg·kg^−1^), manganese (15.4 mg·kg^−1^), copper (1.62 mg·kg^−1^), and zinc (11.4 mg·kg^−1^). The organic carbon content was 28.1%, and total nitrogen was 1.12%, resulting in a C/N ratio of 25:1.

### 4.2. Experimental Variants Used in the Study

The AgNPs concentration of 10.85 mg/L was selected based on prior experimental studies [64] using the same nanoparticle formulation, where this concentration elicited measurable biological responses without inducing acute mortality in non-target organisms. Although average silver nanoparticle concentrations in agricultural soils are typically lower, localized exposure hotspots may occur following repeated applications of nanoparticle-containing agrochemicals, sewage sludge amendments, or accidental releases. Therefore, the applied concentration represents a conservative, upper-bound exposure scenario relevant for ecological risk assessment rather than background environmental conditions.

The aqueous nanoparticle systems used in this study were synthesized without the addition of chemical or biological initiators, external stabilizing agents, or supporting electrolytes, allowing them to be considered matrix-less systems stabilized solely by a cloud of silver cations. Silver nanoparticles (AgNPs) were synthesized in aqueous solutions according to Adamowska et al., 2022 [64]. All preparations were analyzed using SP-ICP-MS. A summary of the most important parameters is shown in Table 1.

The zeta potential of the AgNPs suspension was measured using Zetasizer Nano ZS Size Analyzer (Malvern Panalytical, Worcestershire, UK) yielding a value of −20 mV, indicating colloidal stability and charge characteristics.

The nanoparticle suspension was standardized to approximately 10 mg/L.

Four treatment combinations were tested:

1. Variant 1 (control): 200 cm^3^ of substrate V1, 10 *E. fetida* individuals, and 21 mL of distilled water;
2. Variant 1 (AgNPs): 200 cm^3^ of substrate, 10 *E. fetida* individuals, 20 mL of distilled water, and 1 mL of AgNPs solution at 10.85 mg/L;
3. Variant 2 (control): 200 cm^3^ of compost, 10 *E. fetida* individuals, and 21 mL of distilled water;
4. Variant 2 (AgNPs): 200 cm^3^ of compost, 10 *E. fetida* individuals, 20 mL of distilled water, and 1 mL of AgNPs solution at 10.85 mg/L.

Each condition was replicated three times.

Throughout the experiment, each container received two additions of 8 g of granulated cattle manure: the first at the beginning of the trial and the second after two weeks. The manure used was a granulated product from Den Ouden GrowSolutions (North Brabant, The Netherlands). Additionally, 20 mL of distilled water was added to each container every four days, for a total of seven applications.

After 24 days, mature *E. fetida* specimens exhibiting a visible clitellum were collected from each container and sent for trace element and metabarcoding analyses.

All earthworm individuals from each experimental group were carefully removed from the soil. They were then rinsed with distilled water, gently dried using a paper towel, and weighed. Individuals from each experimental group were flash-frozen with liquid nitrogen and ground to a uniform consistency using a mortar and pestle. The resulting homogenates were placed in sealed tubes and stored at −20 °C until further analysis.

### 4.3. ICP-MS Measurements

Prior to analysis, earthworm samples (0.5 g) were digested using a micro-wave-assisted digestion system (Titan MPS, PerkinElmer, Shelton, CT, USA). Each sample was treated with a mixture of 9 mL of 65% HNO_3_ (Merck SUPRAPUR, Darmstadt, Germany) and 1 mL of H_2_O_2_ (Merck SUPRAPUR) for digestion.

Following the procedure described by Skwiercz et al. [65] elemental analyses were conducted using NexION2200 ICP-MS (PerkinElmer, CT, USA). https://doi.org/10.24925/turjaf.v13i5.1303-1309.7451.

### 4.4. DNA Extraction, Libraries Preparation and Sequencing

Total DNA was extracted using a DNA Mini Kit (Syngen Biotech, Wrocław, Poland), following the manufacturer’s protocol. The V3–V4 regions of the 16S rRNA gene were amplified using the specific primers [66].

The PCR product was purified and a library was constructed using the NEBNext Multiplex Oligos for Illumina 96 Index Primers (New England Biolabs, Salisbury, UK). The resulting PCR products were pooled, and the final purified product was then quantified using qPCR according to the qPCR Quantification Protocol Guide (KAPA LibraryQuantification Kits for Illumina Sequencing platforms, Roche, Basel, Switzerland). The paired-end (2 × 300 bp with V3 chemistry) sequencing was performed using the MiSeq platform (Illumina, San Diego, CA, USA).

Library preparation was performed as described by Zapałowska et al. [67].

The taxa present in the analyzed samples were determined using the metabarcoding approach. The quality of the obtained reads was checked at FastQC [68] and filtered using the Trimmomatic [69] to exclude low-quality reads (Q < 20, sequences with any ambiguous (N) bases, more than six homopolymers). The chimera sequences identified by Mothur 1.31.2 were discarded [70].

Community composition and taxonomic affiliations of the obtained sequences were performed by the software pipeline CCMetagen v1.2.3 [71]. Taxonomic assignment was carried out using NCBI nt data base (accessed 9 December 2025).

The processing and analysis of sequencing data were conducted using the criteria for taxonomic assignment in CCMetagen as follows: species-level similarity threshold of 99.00%, genus-level of 95.00%, family-level of 90.00%, order-level of 85.00%, class-level of 75.00%, and phylum-level of 55.00% [72].

**Figure 5:**
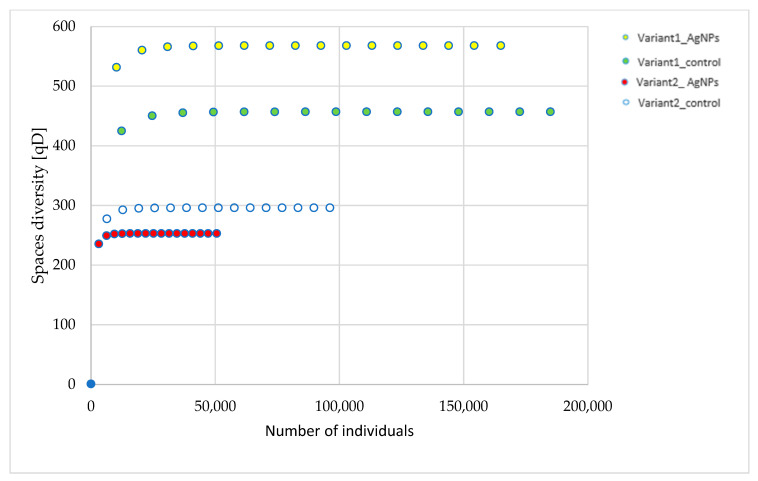
Rarefaction analysis of coverage of OTU detected for different experimental conditions.

Rarefaction analysis was performed to assess sequencing depth adequacy across samples. Rarefaction curves indicated saturation for all experimental conditions, suggesting sufficient coverage of bacterial taxonomic diversity and supporting the use of unrarefied read counts for comparative analyses (Figure 5).

### 4.5. Statistical Analysis

Data were analyzed using Statistica 13.3 (StatSoft Inc., Hamburg, Germany). The normality of data distribution was assessed using the Shapiro–Wilk test, and the homogeneity was verified using Levene’s test. A one-way analysis of variance (ANOVA) was then performed, followed by Fisher’s least significant difference (LSD) and Tukey’s post hoc tests (*p* < 0.05). Pearson correlation coefficients were adjusted using the Bonferroni correction.

## 5. Conclusions

This study highlights how silver nanoparticles (AgNPs) affect *E. fetida* and soil bacterial communities, with effects depending on taxon and substrate type. While some bacteria, such as *Gammaproteobacteria*, *Bacilli*, *Streptococcus*, and *Staphylococcus*, were negatively impacted, *Actinobacteria* and *Bacteroidetes* showed greater tolerance. Compost reduced the inhibitory effects of AgNPs, emphasizing the role of substrate in shaping responses.

The findings underscore the potential of vermicompost and soil organisms to mitigate nanoparticle impacts and support sustainable, circular approaches in agroecosystems, providing essential insights for the safe application of nanotechnology in agriculture.

## Figures and Tables

**Table 1:** Summary of physicochemical characteristics of Ag nanoparticle system employed in this work. The quantification results are associated with an expanded uncertainty of 95% confidence level (number of replicated measurements = 5).

Nanoparticle Size		Number of Nanoparticles (×10^5^)	Concentration of Metal		Total Metal Content (mg/L)
Mean Size (nm)	Most Frequent Size (nm)		In NPs Form (µg/L)	In Ionic Form (mg/L)	
52 ± 1.15	44 ± 0.88	241 ± 42	12.92 ± 0.68	10.84 ± 0.3	10.85 ± 0.3

## Data Availability

The original contributions presented in this study are included in the article. Further inquiries can be directed to the corresponding authors.
